# Cost-Effectiveness and Efficacy of *spa*, SCC*mec*, and PVL Genotyping of Methicillin-Resistant *Staphylococcus aureus* as Compared to Pulsed-Field Gel Electrophoresis

**DOI:** 10.1371/journal.pone.0079149

**Published:** 2013-11-14

**Authors:** Vincent Li, Linda Chui, Lisa Louie, Andrew Simor, George R. Golding, Marie Louie

**Affiliations:** 1 Provincial Laboratory for Public Health, Edmonton, Alberta, Canada; 2 Department of Laboratory Medicine and Pathology, University of Alberta, Edmonton, Alberta, Canada; 3 Department of Microbiology, Sunnybrook Health Sciences Centre, Toronto, Ontario, Canada; 4 National Microbiology Laboratory, Public Health Agency of Canada, Winnipeg, Manitoba, Canada; 5 Provincial Laboratory for Public Health, Calgary, Alberta, Canada; 6 Department of Microbiology, Immunology and Infectious Diseases, University of Calgary, Calgary, Alberta, Canada; University Hospital Münster, Germany

## Abstract

Pulsed-field gel electrophoresis (PFGE) is a valuable molecular typing assay used for methicillin-resistant *Staphylococcus aureus* (MRSA) surveillance and genotyping. However, there are several limitations associated with PFGE. In Alberta, Canada, the significant increase in the number of MRSA isolates submitted to the Provincial Laboratory for Public Health (ProvLab) for PFGE typing led to the need for an alternative genotyping method. In this study, we describe the transition from PFGE to *Staphylococcus* protein A (*spa*), Staphylococcal cassette chromosome (SCC*mec*), and Panton-Valentine leukocidin (PVL) typing. A total of 1915 clinical MRSA isolates collected from 2005 to 2009 were used to develop and validate an algorithm for assigning PFGE epidemic types using *spa*, SCC*mec*, and PVL typing and the resulting data was used to populate a new Alberta MRSA typing database. An additional 12620 clinical MRSA isolates collected from 2010 to 2012 as part of ongoing routine molecular testing at ProvLab were characterized using the new typing algorithm and the Alberta MRSA typing database. Switching to *spa*, SCC*mec*, and PVL from PFGE typing substantially reduced hands-on and turn-around times while maintaining historical PFGE epidemic type designations. This led to an approximate $77,000 reduction in costs from 2010 to 2012. PFGE typing is still required for a small subset of MRSA isolates that have *spa* types that are rare, novel, or associated with more than one PFGE epidemic type.

## Introduction

Methicillin-resistant *Staphylococcus aureus* (MRSA) is widespread in hospital and community settings and has become progressively costly to treat and control [1]–[4]. In Alberta, Canada, MRSA was designated as a “pathogen under surveillance” since June 2005 by Alberta Health, requesting all regional laboratories to submit the first clinical MRSA isolate from each patient within a one year period to the Provincial Laboratory for Public Health (ProvLab) for molecular typing. Routine MRSA genotyping identifies trends in prevalence, distribution, and epidemiology in an effort to enhance patient outcome and reduce transmission. Global dissemination of MRSA is largely attributed to a small number of epidemic MRSA clones that are often predominant in specific geographic regions [5]–[7]. Ten Canadian pulsed-field gel electrophoresis (PFGE) epidemic types (CMRSA 1 to CMRSA 10) have been identified using PFGE by the Canadian Nosocomial Infection Surveillance Program [8], [9].

Although PFGE characterization is useful, it is a labor-intensive and time-consuming technique. Furthermore, PFGE results are prone to subjective interpretation making inter-laboratory comparisons difficult [10]. The number of MRSA isolates submitted annually to ProvLab for genotyping has increased dramatically from 1999 to 2008: with an average of 93 isolates per year in 1999–2004, to an average of 3106 isolates per year in 2005–2008. The difficulties associated with PFGE are enhanced with this increase, making an alternative typing method necessary.

*Staphylococcus* protein A (*spa*) typing is a DNA sequencing assay that assigns *spa* types based on the repeats present in the polymorphic X region of the *Staphylococcus* protein A gene [11]. The National Microbiology Laboratory (NML) in Winnipeg, Canada, uses *spa* typing for MRSA characterization because of the high concordance observed between *spa* types and PFGE epidemic types [12]–[15]. However, some *spa* types correspond to multiple PFGE epidemic types and require additional molecular typing before they can be grouped into a PFGE epidemic type [12]. Golding *et al*., [12] propose that a PCR-based assay to detect the presence of Panton-Valentine leukocidin (PVL) [16] helps to differentiate these strains. Staphylococcal cassette chromosome (SCC*mec*) typing targeting the *mec* gene complex could also further assist differentiation [17], [18].

In this study, we describe the transition from PFGE-based assignment of Canadian PFGE epidemic types to using *spa*, SCC*mec*, and PVL typing. The cost, turn-around times, and assigned PFGE epidemic types using the two different methodologies are compared. This study also evaluates whether SCC*mec* and PVL data can differentiate MRSA isolates that share the same *spa* type but are associated with multiple PFGE epidemic types.

## Materials and Methods

### Bacterial Strains and DNA Extraction

There were three sets of MRSA isolates characterized in this study. The first set includes a selection of 1269 isolates from samples submitted to ProvLab for routine molecular typing between June 2005 and March 2009. These isolates were previously genotyped using PFGE, SCC*mec*, and PVL typing and were selected because they had a unique combination of PFGE, SCC*mec*, and PVL types. The 1269 isolates comprised the validation panel and were *spa* typed and used to populate the Alberta MRSA *spa* typing database. The second set consists of an additional 646 consecutive post-validation clinical isolates received by ProvLab between August 2009 and November 2009. These isolates were *spa*, SCC*mec*, and PVL typed and used for the preliminary evaluation of the developed MRSA typing algorithm. Lastly, ongoing routine MRSA molecular typing continued from January 2010 to December 2012 and included 12620 isolates that were used to determine the efficacy of the typing method as well as the associated time and cost requirements. MRSA isolates were inoculated onto sheep blood agar plates (BAPs; Dalynn Biologicals, Calgary, Alberta, Canada) from frozen cultures for overnight growth at 37°C. DNA was extracted using a modified method described by Holland *et al*. [19]. A single colony was picked from the BAP and suspended in 200 µL of rapid lysis buffer (100 mM NaCl, 10 mM Tris-HCl pH 8.3, 1 mM EDTA pH 9.0, 1% Triton-X), kept frozen at −80°C for 15 minutes, and boiled for 15 minutes. Following cooling at room temperature and centrifugation at 13,000×*g* for five minutes, supernatant was removed and used as DNA template for PCR.

### *spa* Typing

PCR was performed using primers targeting the *spa* gene as described by Golding *et al.* [12]. PCR products from isolates in the validation panel (n = 1269) and post-validation isolates (n = 646) were sent to the Genomic Core DNA facility at the National Microbiology Laboratory (NML) in Winnipeg, Canada, for PCR product cleanup and sequencing. PCR products of isolates that were part of ongoing routine molecular typing (n = 12620) were cleaned and sequenced at ProvLab.

### PFGE, SCC*mec*, and PVL Typing

PFGE was performed as previously described by Mulvey *et al*. [20] using the restriction endonuclease *Sma*I. PFGE results were analyzed using BioNumerics (version 5.1; Applied Maths, USA) and PFGE epidemic type designation was completed based on guidelines detailed by NML and using PFGE fingerprint profiles of reference strains provided by NML [8], [9]. Briefly, isolates were assigned to a PFGE epidemic type if there was a difference of less than seven bands between the PFGE fingerprint pattern of the isolate and a provided reference strain. Isolates were designated as “non-assigned” if they differed by more than seven bands from all reference strains. All isolates were grouped into one of the following PFGE epidemic types: Canadian community-associated MRSA (CA-MRSA) epidemic clones CMRSA 2 (USA800; ST5), CMRSA 7 (USA400; ST1), and CMRSA 10 (USA300; ST8); Canadian hospital-associated MRSA (HA-MRSA) epidemic clones CMRSA 1 (USA600; ST45), CMRSA 2 (USA100, ST5), CMRSA 3/6 (“Punjabi” clone; ST239), CMRSA 4 (USA200; ST36), CMRSA 5 (USA500; ST8), CMRSA 8 (EMRSA-15; ST-22), and CMRSA 9 (no USA equivalent); European epidemic clones (ST88, ST97, and ST80); epidemic clones from the United States USA700 (ST72), USA1000 (China/Taiwan; ST59), and USA1100 (Southwest Pacific(SWP)/Oceania; ST30). SCC*mec* typing was performed using the primers and methods described by Oliveira *et al*. [17]. Epidemic types USA100 and USA800, both characterized as Canadian epidemic type CMRSA 2, were differentiated using SCC*mec* data as only USA800 is SCC*mec* type IV [12], [21]. Isolates that could not be typed using the Oliveria *et al.* protocol [17] were SCC*mec* typed using the method outlined in Kondo *et al*. [18]. There were no isolates in this study that were not typeable by both methods. PVL characterization [16] was performed using previously described methods.

### Data Analysis

Sequencing results for validation and post-validation isolates were obtained from the Genomic Core DNA facility website; sequencing results for isolates part of ongoing routine molecular typing at ProvLab between January 2010 and December 2012 were obtained in-house. All sequences were analyzed in BioNumerics, and submitted to the online Ridom *spa* server (http://www.ridom.de/spaserver), developed by Ridom GmbH and curated by SeqNet.org (http://www.SeqNet.org), for Ridom *spa* type designation [22]. For the validation isolates (n = 1269) that were genotyped using PFGE, SCC*mec*, and PVL typing prior to this study, *spa*-based assignment of PFGE epidemic types was done using databases from NML [12] and Sunnybrook Health Sciences Centre (unpublished data) in Toronto, Canada, that correlate *spa* types with PFGE epidemic types. PFGE epidemic type designations based on *spa* data and PFGE data were compared. Post-validation (n = 646) and ongoing routine molecular typing (n = 12620) isolates were assigned PFGE epidemic types using the Alberta MRSA *spa* typing database and the typing algorithm outlined in this study. Simpson’s Index of Diversity [23] was calculated using the online tool hosted at the Comparing Partitions website (http://darwin.phyloviz.net/ComparingPartitions/).

### Alberta MRSA Typing Database

The genotyping results and PFGE epidemic type designations from the isolates in the validation panel (n = 1269) were used as the initial dataset to build the Alberta MRSA typing database. An algorithm to assign PFGE epidemic types based on the association between *spa*, SCC*mec*, and PVL types with PFGE epidemic types was developed. The database and the algorithm were evaluated using the post-validation subset of isolates (n = 646). PFGE was then performed on a random selection of the post-validation samples to determine if there is a consensus in PFGE epidemic type designation between the different typing methods. Genotyping data from the post-validation isolates and the isolates that were part of routine testing at ProvLab was added to the Alberta MRSA typing database.

### Cost and Time Analysis

The cost and time analysis was based on routine testing of 12620 MRSA isolates submitted to ProvLab for genotyping between January 2010 and December 2012. Values were calculated by averaging the time required for genotyping batches of 20 MRSA isolates during the study period and were rounded to the nearest half hour. Turn-around time calculations began from the isolation of single colonies from overnight cultures and ended when data analysis was complete. Hands-on time was expressed as the sum of the average labor and analysis times required from laboratory technologists.

## Results

### Building the Alberta MRSA Typing Database

The association of *spa*, SCC*mec*, and PVL types with PFGE epidemic types for the validation panel of isolates (n = 1269) that were *spa* typed is shown in Table 1. This genotyping data was used as the foundation for the Alberta MRSA typing database. A total of 160 *spa* types were identified and four of these *spa* types- t008, t044, t1081, and t451 (n = 255; 20% of the genotyped isolates) - corresponded to more than one epidemic type. SCC*mec* typing was needed to assign PFGE epidemic types to t008 (n = 228), which is one of the most common *spa* types observed, because of its association with PFGE epidemic types CMRSA5, CMRSA9, and CMRSA10. The majority of the t008 isolates (n = 226; 99.1%) were characterized as SCC*mec* type IV using primers from Oliveira *et al*., [17] and classified as CMRSA 10. A small number (n = 2) of t008 isolates were not typeable using these primers and could only be SCC*mec* typed using primers from Kondo *et al*. [18]. Both t008 isolates were SCC*mec* type IV and PVL positive and PFGE data was needed to resolve the PFGE epidemic types (one was CMRSA 5; the other was CMRSA 10). PFGE epidemic type classification for *spa* types t044 (n = 17), t1081 (n = 8), and t451 (n = 2) also required PFGE. In total, only 29 of 1269 isolates (2.3%) needed PFGE data for PFGE epidemic type assignment.

**Table 1:** Association of MRSA PFGE epidemic types with *spa*, SCC*mec*, and PVL types in Alberta from June 2005 to March 2009.

PFGE epidemic type	Ridom *spa* type	Kreiswirth repeat succession	SCC*mec*	PVL	Total
CMRSA 1 (USA600)	t004	A2AKEEMBKB	II	−	2
	t026	XKB	IV	−	2
	t065	A2AKBEMBKB	IV	−	9
			V*	−	3
			VI*	−	1
	t1081	XKAX2BMB	IV	−	1
			V*	−	1
	t1082	XKBKAMK	II	−	1
	t116	XKAKEEMBKB	IV	−	2
	t1248	A2AKBEMBKE	IV	−	1
			V*	−	2
	t130	A2BEMBKB	IV	−	1
	t1768	XKAX2BMBMB	IV	−	1
	t230	XKAKB	II	−	1
	t371	A2AKBKB	II	−	1
			IV	−	1
	t5497	A2AK	III	−	1
	t5980	A2AKBBMBBMBBB	IV	−	1
	t715	A2AKBEMB	IV	−	1
	t779	X	IV	−	1
	t865	UJGFMBBB	IV	+	1
	t880	A2AKBB	IV	−	1
CMRSA 1 (USA600) Total					36
CMRSA 2 (USA100)	t002	TJMBMDMGMK	II	−	281
	t003	TMDMGMMK	II	−	88
	t010	TMBMDMGMK	II	−	1
	t014	TMDMGMMMK	II	−	10
	t045	TMDMGMK	II	−	15
	t062	TJMGMK	II	−	2
	t105	TJMBMDMMK	II	−	1
	t111	TJMK	II	−	1
	t1154	TDMGMK	V*	−	7
	t1220	TJMEMDMGMMK	II	−	1
	t1282	TMDMGMMMMK	II	−	2
	t179	TJMBMDMGGK	II	−	1
	t2051	TJMBMDKGMK	II	−	1
	t242	TJMEMDMGMK	II	−	43
	t2958	C3MBMDMGK	V*	−	2
	t306	TJMBMDMGMMK	II	−	7
	t311	TJMBDMGMK	II	−	7
			V*	−	3
	t3234	TJMBME	II	−	1
	t3786	TMDMBMK	II	−	1
	t3948	TGMMK	II	−	1
	t442	C3MBMDMGMK	V*	−	2
	t4695	TJMEMGMK	II	−	1
	t5081	TJMBGMK	V*	−	1
	t548	TJMBMDMGK	II	−	2
	t579	TJMMDMGMK	II	−	1
	t5810	TMDMGMDMGMMK	II	−	1
	t586	TK	II	−	1
	t688	TJMBMK	II	−	2
			V*	−	2
	t985	TJMBMEMGMK	II	−	1
CMRSA 2 (USA100) Total					490
CMRSA 2 (USA800)	t001	TO2MBMDMGMK	IV	−	1
	t002	TJMBMDMGMK	IV	−	14
				+	16
	t003	TMDMGMMK	IV	+	4
	t088	TJMBMDMGGMK	IV	−	3
	t1154	TDMGMK	IV	−	42
	t1781	TKK	IV	−	2
	t179	TJMBMDMGGK	IV	−	2
	t242	TJMEMDMGMK	IV	−	1
	t306	TJMBMDMGMMK	IV	+	1
	t311	TJMBDMGMK	IV	−	33
	t5081	TJMBGMK	IV	−	3
	t539	TJMBMGMK	IV	−	1
	t548	TJMBMDMGK	IV	−	3
				+	1
	t5975	T?	IV	−	1
	t5987	TJMBDJMGMK	IV	−	1
	t688	TJMBMK	IV	−	5
CMRSA 2 (USA800) Total					134
CMRSA 3/6	t037	WGKAOMQ	III	−	60
	t275	WGKAOMQQ	III	−	1
CMRSA 3/6 Total					61
CMRSA 4 (USA200)	t007	WGKKKKAOM	II	−	1
	t012	WGKAKAOMQQ	II	−	4
			IV	−	3
	t018	WGKAKAOMQQQ	II	−	3
	t021	WGKAKAOMQ	IV	−	2
			V*	+	1
	t233	WG	IV	−	1
	t318	WGKKAKAOMQ	IV	+	1
			V*	+	1
	t338	WFKAOMQ	IV	−	1
	t3732	WFKAOMQQ	IV	−	1
	t5976	WGKAAKAOMQQ	IV	−	1
CMRSA 4 (USA200) Total					20
CMRSA 5 (USA500)	t008	YHGFMBQBLO	IV*	+	1
	t064	YHGCMBQBLO	II	−	1
			IV	−	1
	t1677	YHGGMBQBLO	IV	−	1
	t451	YGCMBQBLO	IV	−	1
CMRSA 5 (USA500) Total					5
CMRSA 7 (USA400)	t127	UJFKBPE	II	−	1
			IV	−	5
	t128	UJJFKBPE	II	−	3
			IV	−	20
				+	19
	t1508	WKBPE	IV	−	1
	t175	UJFKKPFKPE	IV	−	2
				+	1
	t1784	UBPE	IV	+	1
	t5469	UJKPE	IV	−	1
	t5475	UMBBPB	II	−	1
	t5977	UJK	IV	+	1
	t5978	UJJDFKBPE	IV	−	1
	t5979	UJFFKPFKPE	IV	+	1
CMRSA 7 (USA400) Total					58
CMRSA 8 (EMRSA-15)	t005	TJEJNCMOMOKR	V*	+	1
	t022	TJEJNF2MNF2MOMOKR	IV	−	5
	t032	TJJEJNF2MNF2MOMOKR	IV	−	11
				+	1
	t2113	TJJEJNF2MNF2MQOKR	IV	−	1
	t223	TJEJCMOMOKR	IV	−	1
			V*	−	1
	t515	TJJEJNF2MNF2MOKKR	IV	−	1
	t578	TJJEJNF2MNF2MOMOR	IV	−	4
	t5982	UJEJNCMOMOKKR	IV	+	2
	t5983	UJENCMOMOKR	IV	+	1
	t852	UJEJNCMOMOKR	IV	+	3
CMRSA 8 (EMRSA-15) Total					31
CMRSA 10 (USA300)	t008	YHGFMBQBLO	IV	−	20
				+	206
			IV*	+	1
	t024	YGFMBQBLO	IV	−	1
			IV	+	3
	t059	YHO	IV	+	1
	t121	YHFMBQBLO	IV	+	1
	t1578	YHGFMBQBM	IV	+	1
	t1635	YHGFMBO	IV	+	3
	t197	YC2BQBLO	IV	+	1
	t211	YHGGFMBQBLO	IV	+	1
	t2792	YHGFMBQBLLO	IV	−	2
	t451	YGCMBQBLO	IV	−	1
	t530	YHGFMBQBK	IV	+	1
	t5989	YHQFMBQBLO	IV	+	1
	t622	YHGFMBLO	IV	+	1
	t818	YHGFMB	IV	−	1
				+	2
	t919	YHGFKBQBLO	IV	−	1
CMRSA 10 (USA300) Total					249
European	t044	UJGBBPB	IV	+	14
	t5984	UJGBBB	IV	+	1
	t5986	UJGB?PB	IV	+	1
European Total					16
Non-assigned	t041	TO2MBMDMBMDMGMK	IV	−	1
	t078	ZFGU2DMGGM	IV	+	1
			V*	−	1
	t084	UJGBBGGJAGJ	II	−	1
			III	−	1
			IV	+	1
	t091	UJFMBGJAGJ	IV	+	1
	t1081	XKAX2BMB	IV	−	2
			V*	−	3
				+	1
	t1379	ZFGMDMGMK	IV	−	1
	t149	TO2MEMDMGMGMK	IV	−	2
	t160	UJFQPLM	II	−	1
			IV	−	1
				+	1
	t164	UG2MFBBLB	IV	+	1
	t1839	TJEFMBBBPB	V*	+	1
	t202	YMJMMKKO	IV	+	2
	t209	UKGJB	II	−	1
	t293	XKAOP2P2P2P2	IV	−	1
	t2982	XMQ	IV	+	1
	t3320	TJEFMBBBQPB	V*	+	2
	t334	YGFMBLO	V*	+	1
	t345	TJEFMBBPB	V*	+	1
	t375	Y2EJCMBPB	II	−	1
	t380	TBPB	IV	−	1
	t405	UBKBPE	II	−	1
	t455	UJGBEPB	IV	+	1
	t525	Y2BJCMBPB	IV	−	1
	t5974	UBEBBBPB	IV	+	1
	t5981	TJBFMBBBQPB	V*	+	1
	t657	TJEFMBPB	V*	+	2
Non-assigned Total					40
ST88	t1816	UGFMBEBBBPB	IV	−	1
	t186	UGFMEEBBPB	IV	−	2
	t6441	UEGFMEBW2EBBBPB	IV	−	1
	t690	UGFMEEBBBPB	IV	+	1
	t692	UGFMBBBBPB	IV	+	3
ST88 Total					8
ST97	t044	UJGBBPB	II	−	1
			IV	−	1
			V*	−	1
	t131	UJGBPB	II	−	1
	t2112	TJGFMBBBBPB	II	−	2
			V*	−	1
	t2297	UJGGFMBBBPB	V*	−	1
	t267	UJGFMBBBPB	IV	−	4
				+	1
	t359	UJGFMBBPB	IV	−	1
	t521	UJGFMBBBBPB	II	−	18
			IV	−	4
	t527	UJGFMBBBBBPB	II	−	2
			IV	−	1
ST97 Total					39
USA1000, China/Taiwan	t163	ZDMDMA3KB	II	−	1
			IV	−	8
	t1751	ZDMDMOE	IV	−	1
	t216	ZDMDMNKB	IV	−	10
				+	2
			V*	−	2
	t2365	ZDKB	IV	−	1
	t316	ZDMNKB	IV	−	1
	t3485	ZDMDMDMOB	IV	+	3
	t437	ZDMDMOB	IV	−	1
				+	2
			V*	+	7
	t441	ZDMOB	IV	−	1
	t4784	ZDDMDMA3KB	V*	−	1
	t976	ZDMDNKB	IV	−	2
USA1000, China/Taiwan Total					43
USA1100, SWP/Oceania	t019	XKAKAOMQ	IV	+	13
	t1133	XKAKAOAOMQ	IV	+	1
	t4341	XKAMQ	IV	+	1
	t5447	UAKAOMQ	IV	+	1
USA1100, SWP/Oceania Total					16
USA700	t126	UJGFMGGM	IV	−	1
	t1346	UJGFGMDMGGGM	IV	−	4
				+	1
	t148	UJGFGMDMGGM	II	−	1
			IV	−	9
	t324	UJGGMDMGGM	IV	−	2
	t4359	UJGGM	V*	−	1
	t537	UJGFGDMGG	V*	−	1
	t791	UJGFGMDMGMM	IV	+	3
USA700 Total					23
Grand Total					1269

Isolates marked with an * were SCC*mec* typed using primers from Kondo *et al.* (18); all other isolates were SCC*mec* typed using primers from Oliveira *et al*. (17).

### PFGE Epidemic Type Assignment using *spa*, SCC*mec*, and PVL Data

**Figure 1:**
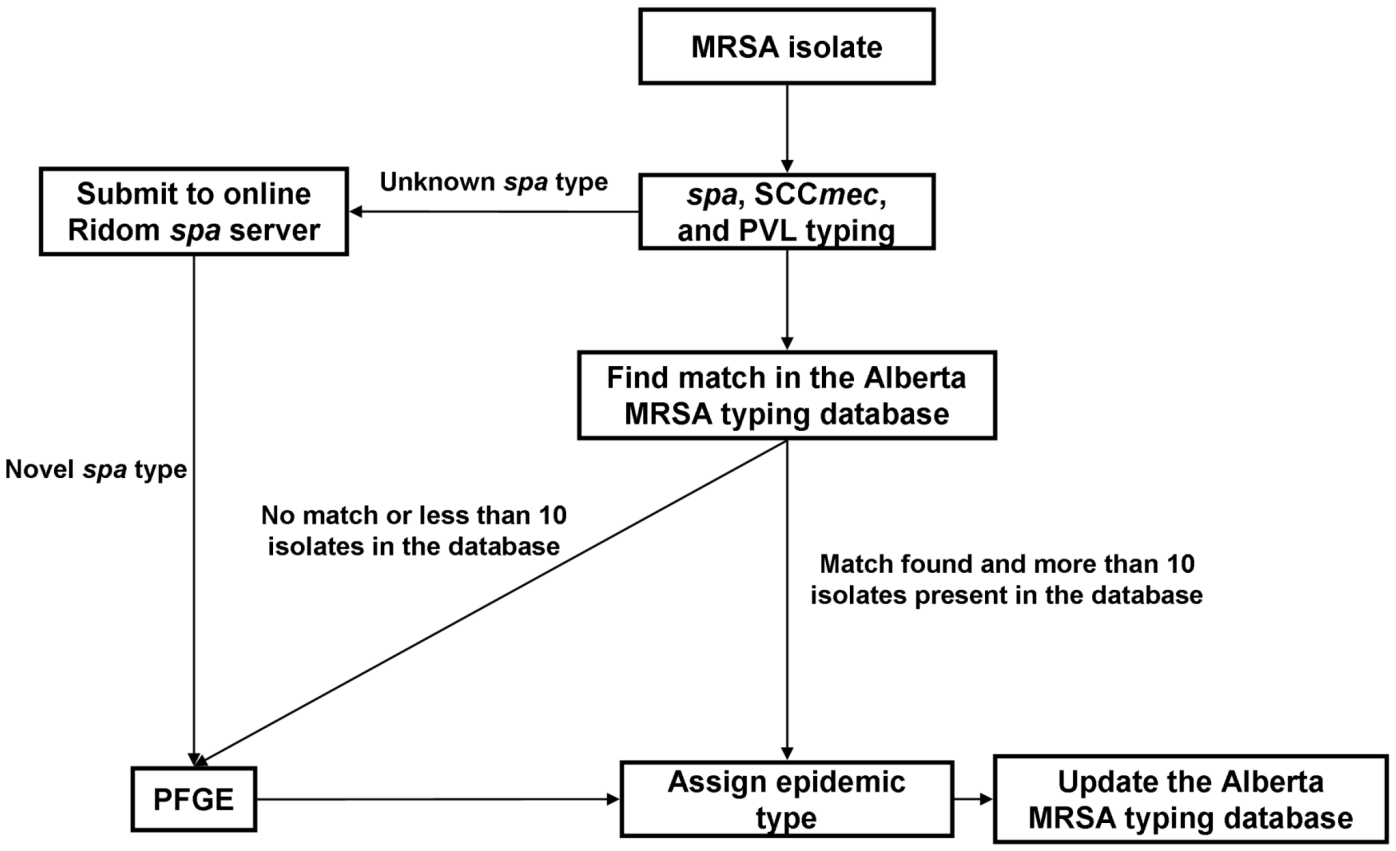
A MRSA genotyping algorithm using *spa*, SCC*mec*, and PVL typing. PFGE epidemic types for MRSA isolates are assigned based on *spa*, SCC*mec*, and PVL data using the Alberta MRSA typing database. PFGE characterization is used for MRSA isolates that have rare or novel spa types, or have *spa*, SCC*mec*, and PVL combinations associated with more than a single PFGE epidemic type.

An algorithm for PFGE epidemic type designation was developed (Figure 1) based on the data generated for the new Alberta MRSA typing database. Uncharacterized MRSA isolates are *spa*, SCC*mec*, and PVL typed. The typing data is then compared to combinations in the new Alberta MRSA typing database: if a match is found and present in sufficient numbers (at ProvLab the arbitrary minimum count is ten isolates) then the PFGE epidemic type is assigned and the database is updated. PFGE epidemic type assignment using PFGE is required for isolates under the following criteria such as 1) no match in the MRSA typing database; 2) rare *spa* types; or 3) typing data combinations that correspond to multiple PFGE epidemic types (Figure 1). PFGE epidemic type designations are then reported as they have been done historically.

A total of 646 consecutive post-validation MRSA isolates, collected from August 2009 to November 2009, were used to evaluate the described MRSA typing algorithm and were assigned PFGE epidemic types using the Alberta MRSA typing database (Table 2). There were 50 *spa* types identified, and two of these *spa* types- t008 (n = 361) and t044 (n = 1) – were associated with multiple epidemic types. The majority (n = 567; 87.8%) of the isolates could be grouped into a PFGE epidemic type using *spa*, SCC*mec*, and PVL data. In particular, SCC*mec* typing data resolved the PFGE epidemic types for 359 of the 361 (99.4%) t008 isolates. PFGE epidemic type designation using PFGE was only needed for 78 isolates (12.1%; Table 2). This group includes isolates with *spa* types corresponding to more than one PFGE epidemic type that cannot be resolved with SCC*mec* and PVL data (n = 3); isolates that have rare *spa* types or ones not previously observed in Alberta (n = 74); and isolates that have novel *spa* types not present in the online Ridom *spa* server (n = 1). One additional isolate with *spa* type t034 was not typeable using PFGE and a PFGE epidemic type was assigned based on existing data in the NML MRSA typing database.

**Table 2:** Association of MRSA PFGE epidemic types with *spa*, SCC*mec*, and PVL types in Alberta from August 2009 to November 2009.

PFGE epidemic type	Ridom *spa* type	Kreiswirth repeat succession	SCC*mec*	PVL	Total
CMRSA 1 (USA600)	t065	A2AKBEMBKB	IV	−	2^b^
			V*	−	2^b^
CMRSA 1 (USA600) Total					4
CMRSA 2 (USA100)	t002	TJMBMDMGMK	II	−	81
	t003	TMDMGMMK	II	−	24
	t014	TMDMGMMMK	II	−	3
	t045	TMDMGMK	II	−	3
	t242	TJMEMDMGMK	II	−	6
	t306	TJMBMDMGMMK	II	−	3^b^
	t3828	TMBMAMGMMK	II	−	1^b^
CMRSA 2 (USA100) Total					121
CMRSA 2 (USA800)	t010	TMBMDMGMK	IV	−	1^b^
	t1154	TDMGMK	IV	−	2
	t179	TJMBMDMGGK	IV	−	1^b^
	t311	TJMBDMGMK	IV	−	1
CMRSA 2 (USA800) Total					5
CMRSA 3/6	t037	WGKAOMQ	III	−	13
CMRSA 3/6 Total					13
CMRSA 4 (USA200)	t012	WGKAKAOMQQ	II	−	1^b^
			IV	−	1^b^
	t021	WGKAKAOMQ	IV	−	1^b^
				+	1^b^
			V*	+	2^b^
CMRSA 4 (USA200) Total					6
CMRSA 7 (USA400)	t127	UJFKBPE	IV	−	1^b^
	t128	UJJFKBPE	IV	−	16
				+	44
	t1786	UJJE	IV	−	1^b^
	t1787	TJJFKBPE	IV	+	7^b^
	t1788	UJJFBPE	IV	+	2^b^
	t4671	UJJFKBPKBPE	IV	+	1^b^
CMRSA 7 (USA400) Total					72
CMRSA 8 (EMRSA-15)	t022	TJEJNF2MNF2MOMOKR	IV	−	8^b^
	t032	TJJEJNF2MNF2MOMOKR	IV	−	2
	t852	UJEJNCMOMOKR	IV	+	1^b^
CMRSA 8 (EMRSA-15) Total					11
CMRSA 10 (USA300)	t008	YHGFMBQBLO	IV	−	11
			IV*	−	1^a^
			IV	+	348
			IV*	+	1^a^
	t024	YGFMBQBLO	IV	+	7^b^
	t051	YHFGFMBQBLO	IV	+	1^b^
	t068	YHHGFMBQBLO	IV	+	3^b^
	t1610	YHGFMBQBBLO	IV	+	1^b^
	t1883	YHGFMBQO	IV	+	1^b^
	t2054	YHGFMBOBLO	IV	+	1^b^
	t211	YHGGFMBQBLO	IV	+	2^b^
	t3081	YHGFMBPO	IV	+	2^b^
	t5989	YHQFMBQBLO	IV	+	2^b^
	t6442	YHGFMBBBLO	IV	+	1^c^
	t723	YHGBLO	IV	+	2^b^
	t818	YHGFMB	IV	+	4^b^
CMRSA 10 (USA300) Total					388
European	t044	UJGBBPB	IV	+	1^a^
European Total					1
ST398	t034	XKAOAOBQO	V*	+	1^d^
ST398 Total					1
ST97	t521	UJGFMBBBBPB	IV	−	1^b^
ST97 Total					1
USA1000, China/Taiwan	t1894	ZDMDMNMOB	V*	+	1^b^
	t437	ZDMDMOB	IV	+	1^b^
	t4784	ZDDMDMA3KB	V*	−	1^b^
	t976	ZDMDNKB	IV	+	1^b^
USA1000, China/Taiwan Total					4
USA1100, SWP/Oceania	t019	XKAKAOMQ	IV	+	13
	t138	XKAOMQ	IV	+	1^b^
USA1100, SWP/Oceania Total					14
USA700	t148	UJGFGMDMGGM	IV	+	1^b^
	t791	UJGFGMDMGMM	IV	−	1^b^
USA700 Total					2
Non-assigned	t3320	TJEFMBBBQPB	V*	+	1^b^
	t657	TJEFMBPB	V*	+	2^b^
Non-assigned Total					3
Grand Total					646

Isolates with *spa* types that correspond to more than one epidemic type and require PFGE for PFGE epidemic type assignment^a^, are rare or have not been previously observed in Alberta^b^, are novel *spa* types^c^, or could not be genotyped using PFGE^d^ are shown. Isolates marked with an * were SCC*mec* typed with primers from Kondo *et al.* (18); all other isolates were SCC*mec* typed using primers from Oliveira *et al*. (17).

### Validation of PFGE Epidemic Types Assigned using *spa*, SCC*mec*, and PVL Typing

The accuracy of PFGE epidemic type assignment using the Alberta MRSA typing database and *spa*, SCC*mec*, and PVL data was assessed by performing PFGE on 49 isolates randomly selected from the post-validation subset of samples. Isolates were then grouped into PFGE epidemic types based on the PFGE fingerprint patterns. A comparison of *spa*-, SCC*mec*-, and PVL-based and PFGE-based epidemic type designation showed that all 49 isolates, representing 10 *spa* types and 20 PFGE fingerprint profiles, shared the same PFGE epidemic type designation regardless of typing method. Simpson’s index of diversity for *spa* typing was 0.853 (95% CI, 0.810–0.896) and increased to 0.859 (95% CI, 0.813–0.905) with the addition of SCC*mec* typing alone or in conjunction with PVL characterization. These values overlapped with Simpson’s index of diversity for PFGE typing (0.918; 95% CI, 0.875–0.960), suggesting the two typing algorithms had similar discriminatory power in this study.

### Distribution of SCC*mec* and PVL Types in Alberta

The distribution of SCC*mec* and PVL types in the set of validation and post-validation isolates is shown in Table 3. Most of the isolates were SCC*mec* typed using primers from Oliveira *et al*., [17] but SCC*mec* typing for some (69 of 1915 isolates; 3.6%) required additional primers [18]. SCC*mec* type IV (n = 1129; 59.0%) was observed most frequently, followed by II (n = 644; 33.6%) then III (n = 76; 4.0%). The majority of these isolates were PVL negative (n = 1101; 57.5%) rather than PVL positive (n = 814; 42.5%). Isolates with Canadian community-associated PFGE epidemic types were predominantly SCC*mec* type IV (902 of 907; 99.4%) and PVL positive (699 of 907; 77.1%). In contrast, isolates with Canadian hospital-associated PFGE epidemic types were mostly SCC*mec* type II (608 of 797; 76.3%) and PVL negative (779 of 797; 97.7%).

**Table 3:** Distribution of SCC*mec* and PVL types in validation and post-validation isolates.

		SCC*mec*/PVL							
		II	III		IV		V		VI
PFGE epidemic type	Numberofisolates	−	−	+	−	+	−	+	−
**CMRSA 1 (USA600)**	40	5 (12.5)	1 (2.5)	0	24 (60.0)	1 (2.5)	8 (20.0)	0	1 (2.5)
**CMRSA 2 (USA100)**	610	593 (97.2)	0	0	0	0	17 (2.8)	0	0
**CMRSA 2 (USA800)**	140	0	0	0	117 (83.6)	23 (16.4)	0	0	0
**CMRSA 3/6**	74	0	73 (98.6)	1 (1.4)	0	0	0	0	0
**CMRSA 4 (USA200)**	26	9 (34.6)	0	0	11 (42.3)	2 (7.7)	0	4 (15.4)	0
**CMRSA 5 (USA500)**	5	1 (20.0)	0	0	3 (60.0)	1 (20.0)	0	0	0
**CMRSA 7 (USA400)**	130	5 (3.8)	0	0	48 (36.9)	77 (59.2)	0	0	0
**CMRSA 8 (E-MRSA15)**	42	0	0	0	33 (78.6)	8 (19.0)	0	1 (2.4)	0
**CMRSA 10 (USA300)**	637	0	0	0	38 (6.0)	599 (94.0)	0	0	0
**European**	17	0	0	0	0	17 (100.0)	0	0	0
**ST88**	8	0	0	0	4 (50.0)	4 (50.0)	0	0	0
**ST97**	40	24 (60.0)	0	0	12 (30.0)	1 (2.5)	3 (7.5)	0	0
**ST398**	1	0	0	0	0	0	0	1 (100.0)	0
**USA700**	25	1 (4.0)	0	0	17 (68.0)	5 (20.0)	2 (8.0)	0	0
**USA1000, China/Taiwan**	47	1 (2.1)	0	0	25 (53.2)	9 (19.1)	4 (8.5)	8 (17.0)	0
**USA1100, SWP/Oceania**	30	0	0	0	0	30 (100.0)	0	0	0
**Non-assigned**	43	5 (11.6)	1 (2.3)	0	10 (23.3)	10 (23.3)	5 (11.6)	12 (27.9)	0
**Total**	1915	644 (33.6)	75 (3.9)	1 (0.1)	342 (17.9)	787 (41.1)	39 (2.0)	26 (1.4)	1 (0.1)

Cell percent values relative to the row total are given in brackets.

### PFGE Epidemic Type Assignment using the Alberta MRSA Typing Database

From January 2010 to December 2012, a total of 12620 first clinical MRSA isolates were submitted to ProvLab for molecular typing and characterized using the Alberta MRSA typing database and the described typing algorithm (Figure 1). The percentage of isolates requiring PFGE for PFGE epidemic type assignment from 2010 to 2012 is shown in Table 4 and decreased from 15.1% to 9.5% during this time period. SCC*mec* typing resolved the PFGE epidemic types for over 99% (n = 5923) of the t008 isolates genotyped (Table 4).

**Table 4:** Routine molecular testing of MRSA isolates using the Alberta MRSA typing database from 2010 to 2012.

Year	Total # of MRSAisolates genotyped	# of MRSA isolatesgenotyped by PFGE	Total # of t008isolates	# of t008 isolates resolvedby SCC*mec* typing
2010	3829	578 (15.1%)	1889	1880 (99.5%)
2011	4306	516 (12.0%)	2021	2012 (99.6%)
2012	4485	427 (9.5%)	2013	2009 (99.8%)

Percentages of total isolates are given in brackets.

### Comparing the Cost and Time Associated with PFGE and *spa*, SCC*mec*, and PVL Typing

The total hands-on time required for PFGE typing (7.5 hours) is greater than the hands-on time required for *spa*, SCC*mec*, and PVL typing (3.5 hours) for 20 strains of MRSA. This is attributed to longer labor and data analysis times (Table 5). In addition, turn-around times are improved for *spa*, SCC*mec*, and PVL typing (10.5 hours) compared to PFGE typing (27.5 hours) as the experiment run times are significantly reduced. Of the 12620 MRSA isolates genotyped between January 2010 and December 2012, 11099 isolates did not require PFGE typing. This reduced the required hands-on time by approximately 2220 hours. Assuming the average salary of a laboratory technologist is $35 per hour, ProvLab saved $77,700 in labor costs which offsets the slightly higher cost of materials associated with *spa*, SCC*mec*, and PVL typing.

**Table 5:** Comparison of time associated with MRSA PFGE and *spa*, SCC*mec*, and PVL typing with times calculated in hours.

	PFGE typing (per 20 isolates)	*spa*/SCC*mec*/PVL typing (per 20 isolates)
Labor time	5.0	3.0
Data analysis time	2.5	0.5
Total hands-on time	7.5	3.5
Total turn-around time	27.5	10.5

## Discussion

Although PFGE is a powerful MRSA genotyping technique, it has many limitations. As the number of MRSA isolates submitted to ProvLab increased, PFGE characterization became much less viable. In this study, we describe the transition from using PFGE to assign MRSA PFGE epidemic types, to using *spa*, SCC*mec*, and PVL typing. With the new Alberta MRSA typing database, most (n = 11666; 87.9%) of the 13266 isolates genotyped after the validation of the typing algorithm were grouped into PFGE epidemic types using *spa*, SCC*mec*, and PVL data. A total of 1600 isolates (12.1%) still needed PFGE for PFGE epidemic type assignment. The number of first clinical MRSA isolates requiring PFGE for PFGE epidemic type assignment steadily declined from 15.1% in 2010 to 9.5% in 2012, further reducing the time and cost associated with MRSA genotyping at ProvLab. As the new Alberta MRSA typing database becomes more populated, isolates with rare or novel *spa* types will become less frequent leading to additional reductions in the PFGE workload.

In this study, isolates with community-associated epidemic types were predominantly SCC*mec* type IV and PVL positive, while those with hospital-associated epidemic types were mostly SCC*mec* type II and PVL negative. These findings are consistent with global trends (as reviewed in [24]). An isolate with *spa* type t034 was observed in this study and it could not be genotyped using PFGE or be assigned to a PFGE epidemic type using the new Alberta MRSA typing database. This isolate was grouped into the PFGE epidemic type ST398 based on past designations made by NML, is known to be resistant to *Sma*I digestion, and is associated with livestock [25], [26]. Characterization of similar isolates in the future will need to be done using only *spa*, SCC*mec*, and PVL data.

PFGE epidemic type designations were consistent regardless of typing method for selected isolates, a result seen in other studies [12], [14], [27], [28], allowing for an easy transition between the two typing methodologies. Although the cost of laboratory supplies for PFGE typing at ProvLab are lower, the cost differential is offset by the significant time and subsequent labor cost savings gained from using *spa*, SCC*mec*, and PVL typing. The turn-around and hands-on times required for *spa*, SCC*mec*, and PVL typing is reduced by more than half compared to PFGE. This is due in part to the computer automation of *spa* typing analyses. At ProvLab, the *spa* typing module accompanying the BioNumerics software is used to automatically analyze *spa* data and assign PFGE epidemic types. In contrast, PFGE data must be manually interpreted and PFGE epidemic types are assigned after careful comparison with representative epidemic strains. As a result, analysis of PFGE data is much more subjective and susceptible to interpretation errors.

SCC*mec* typing was needed to resolve MRSA isolates associated with more than one PFGE epidemic type and was particularly useful for differentiating t008 isolates, as well as PFGE epidemic types CMRSA 2 (USA100) and CMRSA 2 (USA800) which otherwise could not be resolved using PFGE. PVL characterization was not able to differentiate isolates associated with multiple PFGE epidemic types, although it could be used as a general indicator of PFGE epidemic type for select *spa* types (t044). Although SCC*mec* and PVL results are also manually interpreted, these assays produce data that is much faster to analyze.

To conclude, we created a new MRSA typing database in Alberta and validated and used an algorithm for genotyping MRSA using *spa*, SCC*mec*, and PVL typing. The shift away from PFGE typing provides significant time and cost savings, and enables high throughput processing while maintaining historical PFGE epidemic type assignments and reporting. Although PFGE typing is still required at ProvLab, its role is limited and will be further reduced as the Alberta MRSA typing database becomes more populated. A limitation of this study is that it was done at a reference provincial public health laboratory; thus the findings may not be applicable to all settings and other typing methods may still have an important role to play. Additionally, the time and cost savings will vary significantly between different laboratories because of differences in equipment, salary, cost of materials, and technical expertise.
